# Estimating the duration of a central venous catheter at time of insertion

**DOI:** 10.1186/cc14151

**Published:** 2015-03-16

**Authors:** MJ Holmberg, LW Andersen, A Graver, SB Wright, D Yassa, MD Howell, MW Donnino, MN Cocchi

**Affiliations:** 1 Beth Israel Deaconess Medical Center, Boston, MA, USA; 2 University of Chicago, IL, USA

## Introduction

The aim of this study was to investigate whether clinicians can estimate, at the time of insertion, the length of time a central venous catheter (CVC) will remain in place, and to identify clinical variables which may predict CVC duration. CVC-related bloodstream infection is a known complication among critically ill patients. As infection rates may increase with duration of catheterization, more expensive antimicrobial-coated catheters may be used in patients with anticipated long duration of CVC use.

## Methods

We conducted a single-center, prospective study from January 2012 to November 2012. Clinicians prospectively estimated the anticipated duration of CVC at the time of line placement in an electronic procedure note. We collected demographics, past medical history, type of ICU, vital signs, laboratory values, SOFA score, mechanical ventilation and use of vasopressors at the time of placement. Continuous variables were compared with the Wilcoxon rank-sum test and categorical variables with the Fisher's exact test. Pearson's correlation coefficient was used to assess the correlation between estimated CVC time and actual time. Duration of CVC use was dichotomized into long (≥7 days) or short (<7 days), based on previous literature, and sensitivity and specificity for predicting long duration was calculated. We performed a logistic regression analysis to identify variables associated with long CVC duration and calculated the area under the ROC curve (AUC).

## Results

We enrolled 150 patients; median age was 65 (IQR: 52 to 74), 63 (42%) were female and mortality was 22%. Median time from CVC placement to removal was 5 (IQR: 3 to 8) days. The correlation between estimated CVC time and actual time was low (*r *= 0.36, *P *< 0.001). Forty-eight (32%) patients had a long CVC duration. Clinician estimate had 46% sensitivity and 76% specificity for predicting long duration of CVC. Of 30 variables tested, only temperature at the time of insertion was significantly associated with long duration (OR: 1.30, 95% CI: 1.04 to 1.63, *P *= 0.02). The AUC for this model was 0.59 (95% CI: 0.49 to 0.69).

## Conclusion

Our results suggest a low correlation between clinician prediction at time of insertion and actual duration of CVC. We did not find any good predictors of long duration of CVC. Given our relatively low sample size, we may have been underpowered. It may not be feasible to identify patients at the time of insertion who may benefit from antimicrobial-coated catheters.

